# Susceptibility of *Anopheles stephensi* SDA500 strain to common insecticides and efficacy of glazed tile bioassay for resistance characterization

**DOI:** 10.1016/j.crpvbd.2026.100347

**Published:** 2026-01-01

**Authors:** Michele Matera, Melanie Nolden, Sebastian Horstmann, Derric Nimmo, Mark J.I. Paine, David Weetman

**Affiliations:** a Mosquito Management, Envu, 2022 ES Deutschland GmbH, Raiffeisenstr. 16, 40764 Langenfeld, Germany; b Department of Vector Biology, Liverpool School of Tropical Medicine, Pembroke Place, Liverpool, L3 5QA, United Kingdom; c Innovative Vector Control Consortium, Pembroke Place, Liverpool, L3 5QA, United Kingdom

**Keywords:** *Anopheles stephensi*, Glazed tiles, Insecticide screening, Pyrethroid, Resistance, Vector control

## Abstract

Research on the urban malaria vector *Anopheles stephensi* has intensified in recent years following its rapid spread throughout the Horn of Africa and beyond. In addition to behavioural and ecological traits which may limit the efficacy of control efforts, insecticide resistance is a notable problem in invasive *An. stephensi* populations. The most frequently used laboratory reference strain for *An. stephensi* is SDA500 originally colonized from Pakistan; though considered insecticide susceptible, quantitative demonstration of this crucial assumption is lacking. We characterized the susceptibility status of SDA500 against multiple insecticide classes used for adult and larval control using the standard WHO techniques for larval bioassays and two alternatives for adults: bottle bioassays and glazed tile bioassays. SDA500 showed full susceptibility against all insecticides tested, and *via* dose-response assays, we provide the first comprehensive LC_50_ dataset for a strain of *An. stephensi*, filling a key knowledge gap and providing an important resource for all future studies of resistance in this important malaria vector. Whilst tile and bottle adult bioassays produced broadly comparable results for both SDA500 and additional laboratory strains, differences were found when testing neonicotinoids and butenolides, which require the addition of the compound MERO® for effectiveness. Nevertheless, the glazed tile bioassay represents a much higher throughput and less resource-intensive technique than bottle bioassays for simultaneous screening of multiple insecticides.

## Highlights

• The reference SDA500 strain of *Anopheles stephensi* was characterized for susceptibility to 19 insecticides.
• Dose-response curves provided a baseline for future studies on resistance in this invasive malaria vector.
• Two methods – standard WHO bottle bioassay and the newer glazed tile assay were compared.
• Whilst results from the methods were generally correspondent, divergence was noted for insecticides requiring MERO®.
• Glazed tile assays may represent an economical, rapid and higher throughput alternative to bottle bioassays.

## Graphical abstract

**Figure:**
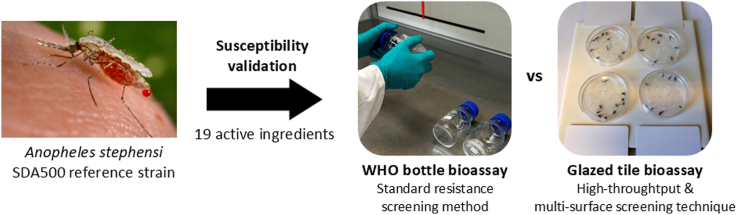

## 1 Introduction

*Anopheles stephensi*, a major malaria vector in Southeast Asia, has become of high interest because of its spread through the Horn of Africa (Sinka et al., 2020). The first reports were in Djibouti in 2012 (Faulde et al., 2014), followed by discovery in Ethiopia and Sudan in 2016 (Carter et al., 2018; Ahmed et al., 2021). Subsequently, the species has been detected in Somalia (Ali et al., 2022), Eritrea (Malaria Threat Map (who.int), Nigeria (NIMR, 2022), Kenya (Ochomo et al., 2023) and Ghana (Afrane et al., 2024). Due to its urban habitat preference, *An. stephensi* is projected to thrive in African cities, with modelling suggesting that over 126 million urban inhabitants could be at risk (Sinka et al., 2020).

The most common control strategies against African malaria vectors are insecticide-treated nets (ITN) and indoor residual spraying (IRS) (Sherrard-Smith et al., 2018), but the more exophilic behaviour of *An. stephensi* than the predominant major African malaria vectors (Manouchehri et al., 1976; Vatandoost et al., 2006; Sinka et al., 2011) is likely to substantially reduce their effectiveness. Studies in the invasive range are currently mostly limited to Ethiopia, where resistance to pyrethroids, carbamates and organochlorines has been found to be common (Yared et al., 2020; Teshome et al., 2023), whilst resistance to organophosphates is more mixed (Balkew et al., 2021; Dugassa et al., 2025). Overall, this pattern largely reflects those found in the native range (Enayati et al., 2020). Although specific mechanisms have rarely been identified, resistance phenotypes appear to be more attributable to metabolic resistance, with both cytochrome P450s and esterases implicated and mutations in the sodium channel and acetylcholinesterase target sites, which are common in *Anopheles gambiae*, rare or absent, respectively (Enayati et al., 2020; Yared et al., 2020). The challenges posed by both behavioural variation and physiological resistance place a premium on rational deployment strategies using effective insecticides (Abiy et al., 2025).

Possible alternative control strategies against *An. stephensi* might exploit the propensity of *An. stephensi* to occupy urban habitats and breed in stored water containers, which may be extensively used during the dry season (Thomas et al., 2016; Ahmed et al., 2022; Hemming-Schroeder and Ahmed, 2023), and are potentially far more readily identified than typical rural *Anopheles* breeding sites. Larval source management (LSM) techniques, whereby breeding habitats are modified, manipulated, or treated, usually with insecticides (WHO, 2012), are therefore considered a promising option to combat *An. stephensi* (Hemming-Schroeder and Ahmed, 2023), especially if the most productive habitats can be identified and prioritized for control (Yared et al., 2025). For adult bioassays, diagnostic doses are available for many, though not all, insecticides, whilst for larvae dose-response curve protocols are more commonly employed. Dose-response assays provide much richer data capable of quantifying resistance levels, rather than prevalence estimates provided by diagnostic dose bioassays (Lees et al., 2022), with levels typically represented as a resistance ratio, usually of 50% lethal concentration (LC_50_) values, between the strain of interest and a fully susceptible laboratory colony. This sensitive, quantitative method of quantifying resistance depends crucially on the availability of a laboratory colony proven to be susceptible to insecticides. Surprisingly, given their importance, comprehensive multi-insecticide datasets for laboratory strains defined as susceptible are rare.

With a pressing need to develop new control measures, which may involve new or seldom-previously-used insecticides against *An. stephensi*, it is important to establish a well-characterised susceptible line to be used as a susceptible reference point. The *An. stephensi* SDA500 strain has been used in many studies around the world, constituting a reference for multiple purposes, including insecticide resistance, genomics and transcriptomics studies (Neafsey et al., 2015; Chida et al., 2020; Zhong et al., 2024), as well as immunity studies (Couto et al., 2017) and CRISPR-based gene editing (Larrosa-Godall et al., 2025). The SDA500 strain was first established in 1982, *via* selection for increased susceptibility to *Plasmodium falciparum* from a previously established mixed-stock strain originating from the Sind Province, Pakistan, reared by the PMRC (Pakistan Medical Research Centre) (Feldmann et al., 1990; Feldmann and Ponnudurai, 1989). Though considered susceptible owing to its long time since colonization, no quantitative information is available in the literature about the insecticide susceptibility status of the strain, despite its use as a reference.

Provision of sufficient data for accurate estimation of dose-response curves is challenging for adult mosquitoes, especially when there is a need to simultaneously test multiple insecticides or populations. The primary methodology used is the bottle bioassay, in which 250 ml glass Wheaton bottles are coated internally with insecticide (dissolved in acetone) at a range of concentrations. In some cases, for example with neonicotinoids and the butanolide flupyradifurone, MERO® (rapeseed oil methyl ester 81%, RME) is added to improve insecticide performance (WHO, 2022b). Though well standardised and widely applied, the bottle bioassay method is resource- and labour-intensive. The specified bottles are quite expensive, large and heavy, and the preparation procedure ideally uses a mechanical roller to evenly coat the insecticide mixture.

The glazed tile bioassay is a more recently developed method, which relies on the same principle of insect tarsal contact for insecticide uptake as the WHO bottle assay (as well as the widely used WHO tube assay) (WHO, 2022b). Multiple early studies on insects other than mosquitoes used a range of tile materials and insecticide application methods (McLaren et al., 1986; Wege et al., 1999; Toews et al., 2003; Barile et al., 2008; Jones and Bryant, 2012). A mosquito-specific protocol was developed by Horstmann and Sonneck (2016) using ceramic tiles manually coated with insecticides dissolved in acetone, and subsequently employed by Nolden et al. (2021). This glazed tile methodology requires fewer and cheaper consumables, which do not necessarily require a specific supplier (standard-sized glazed wall tiles *vs* Wheaton bottles) and a quicker preparation, with tiles drying far faster than the 24 h required for bottles. Whilst presenting significant advantages in cost and throughput, the glazed tile bioassay has never been validated by cross-comparison with WHO-recommended adult mosquito bioassays, an essential step in a formal validation process (Matope et al., 2023).

Here, as part of work to quantitatively characterise the susceptibility status of the SDA500 *An. stephensi* colony against a wide range of insecticides, we compared the relative performance of the glazed tile and bottle bioassay methodologies for SDA500 and also additional laboratory colonies of other *Anopheles* malaria vector species.

## 2 Materials and methods

### 2.1 Mosquito strains

The experiments were performed on four *Anopheles* strains reared in Envu laboratories (Monheim, Germany): SDA500 (*An. stephensi*); Tiassalé-S (*An. gambiae*); FANG (*An. funestus*); FUMOZ-R (*An. funestus*) (Table 1).

**Table 1:** Mosquito strains involved in the study, with information about origin and susceptibility.

Species	Strain	Susceptibility	Origin	Obtained from	Date acquired
*An. stephensi*	SDA500	Susceptible	Pakistan	University of Heidelberg	2022
*An. gambiae*	Tiassalé-S	Susceptible	Côte d’Ivoire	LECA	2021
*An. funestus*	FANG	Susceptible	Angola	LITE	2019
*An. funestus*	FUMOZ-R	Pyrethroid-resistant	Mozambique	NICD	2011

*Abbreviations*: LECA, Laboratoire d’Ecologie Alpine (Grenoble, France); LITE, Liverpool Insect Testing Establishment (Liverpool, UK); NICD, National Institute for Communicable Diseases (Johannesburg, South Africa).

Mosquitoes were reared in controlled conditions (27 ± 1 °C temperature, 65 ± 5% relative humidity, 12/12 L:D photoperiod with 1 h dusk/dawn). Eggs were hatched in demineralised water, and emergent larvae fed daily with TetraMin® fish flakes until reaching the pupal stage. Pupae were transferred to cages (46 × 33 × 20 cm) before adult emergence. Adults were fed *ad libitum* through sponges soaked with a 10% dextrose water solution, with blood meals (bovine blood, obtained from Elocin Laboratory, Oberhausen, Germany) provided every two weeks following standard protocols (Das et al., 2007).

### 2.2 Insecticides used

Abamectin (CAS: 71751-41-2), bendiocarb (CAS: 22781-23-3), clothianidin (CAS: 210880-92-5), deltamethrin (CAS: 52918-63-5), diflubenzuron (CAS: 35367-38-5), flupyradifurone (CAS: 951659-40-8), imidacloprid (CAS: 13826-41-3), piperonyl butoxide (PBO) (CAS: 51-03-6), permethrin (CAS: 52645-53-1), pirimiphos-methyl (CAS: 29232-93-7), pyriproxyfen (CAS: 95737-68-1), temephos (CAS: 3383-96-8), transfluthrin (CAS: 118712-89-3), triflumizole (CAS: 68694-11-1) and triflumuron (CAS: 64628-44-0) were obtained from Sigma Aldrich/Merck (Darmstadt, Germany). Alpha-cypermethrin (CAS: 67375-30-8), cis-permethrin (CAS: 61949-76-6) and trans-permethrin (CAS: 61949-77-7) were purchased from Dr Ehrenstorfer (LGC group, Teddington, UK). 4′OH deltamethrin (CAS: 66855-89-8) was synthetised by Bayer AG (Leverkusen, Germany). 4′OH permethrin (CAS: 67328-58-9; ≥ 97%) was synthetised by Aragen (formerly GVK Bio, Hyderabad, India) (Table 2). All chemicals were of analytical grade unless otherwise stated. MERO®, made of 81.4% w/w rapeseed oil methyl ester, was supplied by Bayer CropScience (Monheim, Germany). The anionic and nonionic surfactant Atlox™ 3467 was obtained by Croda Agriculture (Edison, New Jersey).

**Table 2:** Active ingredients involved in the research with related CAS number, type of vector control product including them and discriminating dose (DD) for bottle assay for *Anopheles stephensi* (when available).

Active ingredient	CAS number	Product types	DD for bottle assay
4′OH deltamethrin	66855-89-8^1^	–	–
4′OH permethrin	67328-58-9^2^	–	–
Abamectin	71751-41-2^3^	SS	–
Alpha-cypermethrin	67375-30-8^4^	IRS, ITN	0.0125 mg/ml
Bendiocarb	22781-23-3^3^	IRS	0.0125 mg/ml
Cis-permethrin	61949-76-6^4^	–	–
Clothianidin	210880-92-5^3^	IRS	0.01 mg/ml
Deltamethrin	52918-63-5^3^	ITN, IRS, SS	0.0125 mg/ml
Diflubenzuron	35367-38-5^3^	Larvicide (IGR)	–
Flupyradifurone	951659-40-8^3^	SS	0.06 mg/ml
Imidacloprid	13826-41-3^3^	SS	–
Piperonyl butoxide	51-03-6^3^	Synergist (ITN)	–
Permethrin	52645-53-1^3^	ITN	0.0215 mg/ml
Pirimiphos-methyl	29232-93-7^3^	IRS, larvicide	0.02 mg/ml
Pyriproxyfen	95737-68-1^3^	Larvicide (IGR)	–
Temephos	3383-96-8^3^	Larvicide	–
Transfluthrin	118712-89-3^3^	SS, SRE	0.002 mg/ml
Trans-permethrin	61949-77-7^4^	–	–
Triflumizole	68694-11-1^3^	Synergist	–
Triflumuron	64628-44-0^3^	Larvicide (IGR)	–

*Abbreviations*: IGR, insect growth regulator; IRS, indoor residual spray; ITN, insecticide-treated net; SRE, special repellent emanator; SS, space spray.

*Suppliers: ^1^Bayer AG (Leverkusen, Germany); ^2^Aragen (formerly GVK Bio, Hyderabad, India); ^3^Sigma Aldrich/Merck (Darmstadt, Germany); ^4^Dr Ehrenstorfer (LGC group, Teddington, UK).*

### 2.3 Bioassays

Glazed tile bioassays were used to evaluate insecticide efficacy on adult non-blood-fed female mosquitoes, following the procedure described by Horstmann and Sonneck (2016). Groups of 10 mosquito female adults aged 2–3 days were sorted into perforated Petri dishes and exposed for 30 min to acetone-based active ingredient (a.i.) solutions, with concentrations ranging from 10 mg a.i./ml to 1.02 × 10^−6^ mg a.i./ml. For each mosquito pool, 1125 μl of solution was spread onto the surface of a glazed tile (15 × 15 cm, ceramic, Vitra, Germany) and left to dry for 60 min, obtaining concentrations ranging from 500 mg a.i./m^2^ to 0.0000512 mg/m^2^. Uncoated tiles and acetone-only tiles (treated with 1125 μl acetone) were used as controls. Neonicotinoids (clothianidin, imidacloprid) and flupyradifurone require to use of a solution of acetone with MERO® at a concentration of 2000 ppm for the standard bioassays. Subsequent tests to investigate the effect of MERO® concentration on clothianidin lethality used two additional MERO® concentrations, 200 and 800 ppm. Testing of dispersant effect was performed by dissolving 1% of Atlox™ 3467 in acetone + MERO® solutions before coating the tiles. After 4 h, cotton pads soaked with 10% dextrose water solution were placed onto the tiles to keep the mosquito fed and alive during the experiment. Mortality was assessed 24 h after the exposure.

Synergist assays followed the protocol reported by Nolden et al. (2021), involving a pre-exposure step to PBO or triflumizole before the normal insecticide exposure to deltamethrin. PBO and triflumizole acetone solutions with fixed concentration (0.4 mg a.i./ml) were spread on the glazed tiles and left for 30 min to dry under a fume hood (resulting in a final surface concentration of 40 mg a.i./m^2^). Mosquitoes were then moved onto the tiles and exposed to PBO and triflumizole for 30 min, followed by movement onto deltamethrin-treated glazed tiles for a further 30 min, with subsequent mortality assessment as described above.

WHO bottle bioassays were also used to evaluate the susceptibility status of SDA500 *An. stephensi* colony on adult female mosquitoes, following standard procedures (WHO, 2022b). The only adaptation was a reversion of the WHO recommended drying time of 24 h to the original, and more practical, Centers for Disease Control and Prevention (CDC) bottle bioassay guideline of 1 h (CDC, 2012); optical microscopy showed complete evaporation of acetone after 1 h, supporting the decision to adopt the same drying times for the bottle and glazed tile bioassays. Approximately 25 non-blood-fed female mosquitoes aged 2–3 days were inserted into each bottle using a glass nozzle attached to a mechanical aspirator for a 1 h exposure period, then transferred to paper cups (440 ml; 10 × 9.5 cm) covered with metal mesh. Knockdown levels were recorded at the end of the 1-h exposure, then all the cups were provided with 10% dextrose water solution-soaked cotton pads. Mortality was assessed 24 h after the exposure.

Larval bioassays followed WHO guidelines (WHO, 2005). To test traditional larvicides, 20 fourth-instar larvae were added to a mixture of 100 ml demineralised water and 100 ml breeding water from *An. stephensi* rearing trays. Insecticide solutions were derived from an acetone-based stock solution with subsequent dilutions in water. After the setup of the experiment, all the cups containing larvae were fed with TetraMin® fish flakes; mortality was assessed 24 h after the exposure by counting dead or moribund larvae. To test insect growth regulators, the experimental setup was the same as traditional insecticides except for the use of third-instar instead of fourth-instar larvae to allow a first moulting stage before turning into pupae. Food was provided every day before the development of pupae, and evaporated water was replenished every two days by the addition of 20 ml of demineralised water. Cups with pupae were covered to prevent adults from escaping. Once all surviving immatures emerged as adults, the number of fully developed adults, dead larvae and dead pupae was recorded.

Topical application for clothianidin (alone) followed the WHO protocol (WHO, 2006), adapted in a way similar to Uragayala et al. (2015). Insecticidal solutions with decreasing concentrations (1000–0.1 ppm) were prepared by dissolving clothianidin in acetone. Pools of 10 three-day-old adult females were separated after immobilizing them for 1 min in a freezer and then were kept on a cooling plate at −2 °C to perform the topical applications. The average mosquito weight was obtained in advance by weighing all the individuals involved in the test. Five replicates for each insecticide concentration, plus five replicates of the untreated control, were prepared. A volume of 0.2 μl of insecticide solution was injected onto the pronotum of thorax of each mosquito using a micropipette. Each pool of mosquitoes was placed in a 250 ml-volume plastic cup with a perforated plastic lid (to allow ventilation) and a piece of filter paper soaked in 10% sucrose solution. Knockdown was evaluated 1 h after the exposure, and mortality after 24 h.

Glazed tile bioassays were performed in triplicate; WHO bottle bioassays involved five replicates for each condition. Mortality data obtained from the four types of tests were used to calculate concentration-response curves and, consequently, 50% lethal concentration (LC_50_) values using GraphPad Prism v10.1.2. Mortality levels for each insecticide concentration were corrected where mortality in controls was between 5% and 20% (Abbott, 1925); in case of control lethality over 20%, data were discarded. Curves were fitted to the data, and LC_50_ values (with 95% confidence intervals) calculated using GraphPad Prism. The presence of outliers was assessed using Grubb’s test (https://www.graphpad.com/quickcalcs/grubbs1/) with significance level of *P* < 0.01 to account for multiple testing caused by sequential removal of significant outliers.

The susceptibility status of *An. stephensi* SDA500 was assessed at the discriminating doses (DDs) specified by WHO for bendiocarb, clothianidin, deltamethrin and transfluthrin (WHO, 2022a; Corbel et al., 2023), implemented with DDs recommended by the (WHO, 2022a; Corbel et al., 2023). Equations describing the dose-response curves were used to calculate the mortality levels corresponding to DDs. Standard WHO thresholds were then applied: susceptible as mortality ≥ 98%; suspected resistance (90% ≤ mortality < 98%); resistant (mortality < 90%). For comparison, the same DD levels were used to assess lethality at the corresponding concentrations of insecticides in glazed tiles bioassays.

LC_50_ values (reported in a.i. mass/surface measure unit mg/m^2^) from bottle assays (considering the bottle’s internal surface equal to 0.028 m^2^) were compared to those from glazed tile assays across insecticides using both Pearson and Spearman rank correlations *via* GraphPad Prism. The same approach was followed to compare LC_50_ values of SDA500 with *An. gambiae* Tiassalé-S, *An. funestus* FANG and *An. funestus* FUMOZ-R strains.

### 2.4 Microscopy

Coating differences among different surfaces with and without surfactants dissolved in acetone (or acetone alone) were observed using a compound microscope with Keyence VH-Z100R lenses. The comparison involved the ceramic tiles used for the glazed tile bioassay, and glass tiles of the same size. Both groups of tiles were coated with acetone, acetone + MERO®, clothianidin dissolved in acetone, clothianidin dissolved in acetone + MERO®. All coatings were performed according to the glazed tile bioassay method above. The concentration of clothianidin was 20 mg/m^2^ in all conditions. MERO® was added to acetone in a concentration of 2000 ppm.

## 3 Results

### 3.1 Characterization of insecticide susceptibility of *An. stephensi* SDA500

**Fig. 1:**
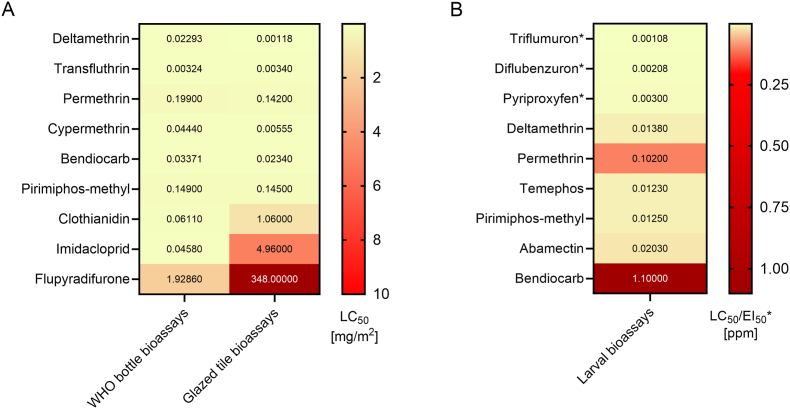
**A** Heatmap reporting mean lethal concentration 50 (LC_50_) values of *An. stephensi* SDA500 strain using glazed tile bioassays and WHO bottle bioassays. **B** Heatmap reporting mean LC_50_ and EI_50_ values of *An. stephensi* SDA500 strain using WHO larval bioassays.

Glazed tiles bioassay was used to obtain a comprehensive characterization of the susceptibility levels of SDA500 against 15 active ingredients (Fig. 1A, full data in Supplementary Table S1). Deltamethrin was the most toxic active ingredient (LC_50_ = 0.0012 mg/m^2^), and permethrin was the least effective pyrethroid (LC_50_ = 0.142 mg/m^2^), with its LC_50_ falling between that of its component isomers, cis-permethrin (LC_50_ = 0.0462 mg/m^2^) and trans-permethrin (LC_50_ = 0.845 mg/m^2^). The primary metabolites of deltamethrin (4′OH deltamethrin) and permethrin (4′OH permethrin) showed higher LC_50_ values compared to the parental compounds (146-fold and 66-fold, respectively). Of all the insecticides, flupyradifurone required by far the highest concentration for effective killing (LC_50_ = 348.0 mg/m^2^).

Data from WHO bottle bioassays were also used to plot dose-response curves and calculate LC_50_ values for a selection of the insecticides against SDA500 female adults (Fig. 1B). Data expressed in mg/ml were converted to mg/m^2^ for comparison with LC_50_ values obtained for glazed tiles bioassays (Fig. 1). Deltamethrin and transfluthrin proved to be the most toxic insecticides (LC_50_ = 0.0029 mg/m^2^ and 0.0032 mg/m^2^, respectively), while again flupyradifurone required the highest concentration (LC_50_ = 1.93 mg/m^2^).

In larval assays, the efficacy of standard insecticides (abamectin, bendiocarb, deltamethrin, permethrin, pirimiphos-methyl, temephos) is reported as mean LC_50_ values expressed in ppm, whilst IGRs (diflubenzuron, pyriproxyfen, triflumuron) are expressed as mean EI_50_ values, reflecting the level of adult emergence inhibition (Fig. 1B; full data in Supplementary Table S2). Among the standard insecticides, temephos was the most toxic (LC_50_ = 0.0123 ppm), while bendiocarb was the least toxic (LC_50_ = 1.1 ppm). IGR toxicity values were all lower than standard insecticides (triflumuron EI_50_ = 0.00108 ppm; diflubenzuron EI_50_ = 0.00208 ppm; pyriproxyfen EI_50_ = 0.003 ppm).

### 3.2 Comparison of adult bioassay techniques

**Fig. 2:**
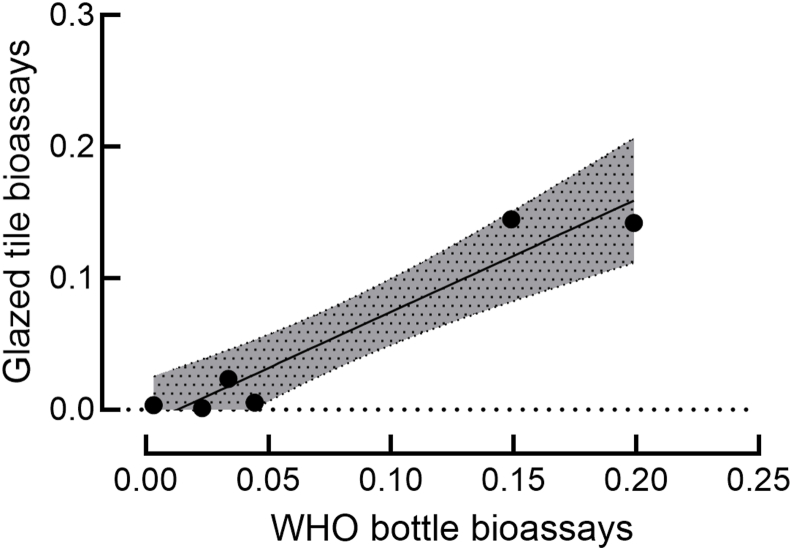
Comparison of WHO bottle bioassays and glazed tile bioassays. Plot of mean LC_50_ values from WHO bottle bioassays and glazed tile bioassays after removal of significant outliers (see text).

LC_50_ values obtained with WHO bottle bioassays and glazed tiles bioassays were significantly correlated (Pearson’s *r* = 0.99, *n* = 9, *P* < 0.001). However, the correlation appeared to be driven by the point for flupyradifurone (Fig. 2), with the LC_50_ for both bottles and glazed tiles a significant outlier (Grubb’s test *P* < 0.01 for each), albeit a far less extreme one in the bottle bioassays (note x and y axis scale differences in Fig. 2). Exclusion of flupyradifurone resulted in a non-significant correlation (*P* = 0.772). Exclusion of the two neonicotinoids (clothianidin and imidacloprid), both of which were also significant outliers in the glazed tile assays (*P* < 0.01), though not in the bottle bioassays, returned a significant Pearson correlation (*r* = 0.963, *n* = 7, *P* = 0.002; Fig. 2B). Notably each of the outlying LC_50_ values in the glazed tile assays involved insecticides co-dissolved with MERO®.

### 3.3 Investigating the differential effects of MERO®

The different behaviours of insecticides requiring MERO® between the two adult bioassay techniques were first investigated by using reduced concentrations of MERO® on the glazed tiles and the bottles for clothianidin. In the glazed tile assays, 800 ppm and 200 ppm MERO® increased the LC_50_ values for clothianidin by 44.2-fold and 141-fold, respectively, compared to the standard concentration of 2000 ppm MERO®. In the bottles, the same concentrations produced much smaller increases in LC_50_ values of 4-fold and 7.55-fold, respectively (Supplementary Fig. S2). These results indicate that the glazed tile assay results are much more sensitive to MERO® concentration than the bottle bioassays.

The possible cause of this differential sensitivity was further explored by testing whether the different materials of glazed tiles (ceramic) and bottles (glass) might lead to variation in effects, by changing the tile substrate to glass in bioassays using clothianidin and flupyradifurone. This hypothesis was not supported: clothianidin proved less effective on glass than ceramic tiles, while flupyradifurone performed only slightly better (LC_50_ = 286.7 mg/m^2^ on glass and 348 mg/m^2^ on ceramic) (Supplementary Fig. S3).

A second hypothesis to explain the differential MERO® effect was that the manual method of coating tiles was causing greater heterogeneity in insecticide dispersion compared to bottle assays. For this, the surfactant Atlox™ (at a 1% concentration) was added to the acetone + MERO® solutions to improve dispersion. This produced contrasting effects, a 3-fold increase in clothianidin’s LC_50_ and a 3-fold decrease in flupyradifurone’s LC_50_ (Supplementary Fig. S4), suggesting that greater heterogeneity resulting from manual dispersion of mixtures was not a general explanation for the high glazed tile assay LC_50_s.

**Fig. 3:**
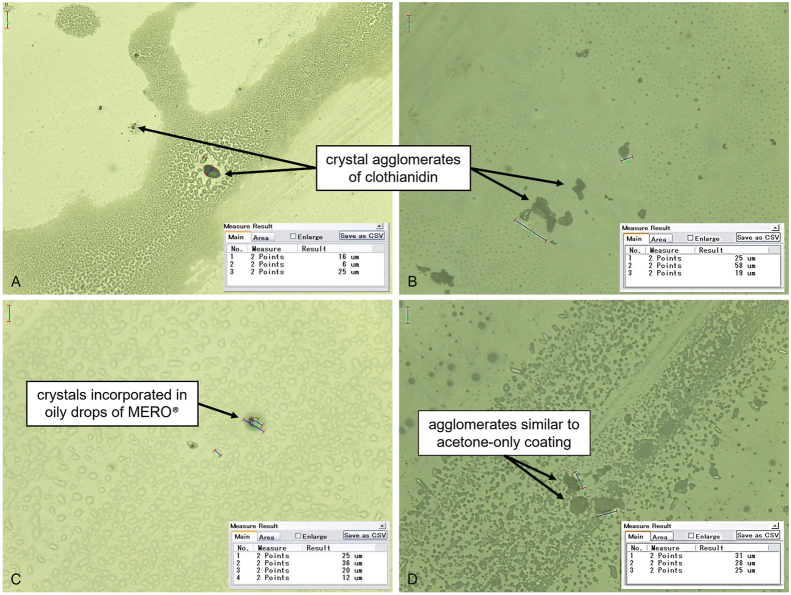
Microscopic images at 500× magnification. **A** Clothianidin dissolved in acetone on glass tiles. **B** Clothianidin dissolved in acetone on glazed tiles. **C** Clothianidin dissolved in acetone + MERO® on glass tiles. **D** Clothianidin dissolved in acetone + MERO® on glazed tiles.

Finally, we visualised whether there were differences in surface presentation of insecticide between tiles of contrasting materials using optical microscopy. Ceramic or glass tiles were coated with: (i) acetone alone; (ii) acetone + 2000 ppm MERO®; (iii) clothianidin dissolved in acetone + 2000 ppm MERO®. There were no traces of acetone (applied without MERO®) after the drying time. MERO® formed a similar pattern on both glass and ceramic, with oily structures visible on each tile type after the 1-h drying time. Clothianidin was visible as crystal agglomerates, which were larger on ceramic than glass tiles (Fig. 3A *vs* 3B). Clothianidin dissolved in acetone + MERO® showed a reduction in the size of the fragments, which were mostly incorporated into the MERO® oily structures. This pattern was more readily observable on glass than on ceramic (Fig. 3C *vs* 3D) and thus might be a possible contributory factor to the differential effects of MERO®.

### 3.4 Comparison of discriminating doses from bottle and tile bioassays

Lethality levels estimated from dose-response curves at the concentration specified as the diagnostic doses by WHO for bottle assays (Vatandoost et al., 2019; WHO, 2022b; Corbel et al., 2023) are shown in Table 3, alongside those for the glazed tile bioassay. For bottle bioassays, the resulting estimates were above the susceptibility threshold (98%) for α-cypermethrin, bendiocarb, deltamethrin and transfluthrin, deltamethrin and bendiocarb. However, the estimate would classify clothianidin and flupyradifurone, applied within the recommended 800 ppm MERO® concentration (WHO, 2022b), as resistant. For clothianidin, this improved to near the susceptibility threshold with addition of 2000 ppm MERO®, the concentration used for glazed tile assays. Pirimiphos-methyl had a predicted lethality classified as suspected resistance (95.5%), while permethrin would be unexpectedly classified as resistant (84%).

**Table 3:** Evaluation of susceptibility through WHO bottle assay and glazed tile assay.

Compound	WHO bottle assay			Glazed tile assay		
	DD (mg/ml)	Predicted lethality at DD (%)	Classification	DD (mg/m^2^)	Predicted lethality at DD (%)	Classification
α-cypermethrin	0.0125	98.5	S	0.446	100	S
Deltamethrin	0.0125	99.4	S	0.446	99.7	S
Permethrin	0.0215	84.0	R	0.767	95.4	SR
Transfluthrin	0.002	99.9	S	0.071	94.4	SR
Bendiocarb	0.0125	99.3	S	0.446	99.3	S
Pirimiphos-methyl	0.02	95.5	SR	0.714	95.6	SR
Clothianidin (MERO® 800 ppm)	0.01	71.8	R	0.357	0.03	R
Clothianidin (MERO® 2000 ppm)	0.01	97.7	SR	0.357	11.6	R
Flupyradifurone (MERO® 2000 ppm)	0.06	54.1	R	2.14	10.9	R

*Abbreviations*: DD, discriminating dose; S, susceptible; R, resistant; SR, suspected resistance.

The DDs proposed for bottle assays were adapted to glazed tiles bioassays by conversion to mg/m^2^, maintaining the same mg a.i./surface area ratios. Results were very similar to those from the bottle bioassays for α-cypermethrin, bendiocarb, deltamethrin and pirimiphos-methyl, improved for permethrin, classified as suspected resistance, slightly lower for transfluthrin, though this would still not correspond to a resistant phenotype, and, as expected from previous results (above), dramatically lower for clothianidin, whether the concentration of MERO® in acetone was at 800 ppm or 2000 ppm, and flupyradifurone (Table 3).

To provide a reference point for future studies we also performed the topical application method (WHO, 2006) with clothianidin on *An. stephensi* females. No diagnostic dose for the topical application method is available, but the resulting LC_50_ value of 0.0872 μg a.i./mg may serve as a guideline denominator for future calculations of resistance ratios for strains of unknown status.

Finally, to determine whether the most common metabolic resistance mechanism for pyrethroids (elevated P450 activity) might be present in SDA500, we performed synergist assays with either for PBO and triflumizole prior to deltamethrin exposure (Supplementary Fig. S5). Confidence intervals for deltamethrin alone or in combination with either synergist overlapped at every concentration on the dose-response curve, indicating no significant effect of P450 metabolic mechanisms on the phenotype.

### 3.5 Comparison of glazed tile bioassays among *Anopheles* strains

**Fig. 4:**
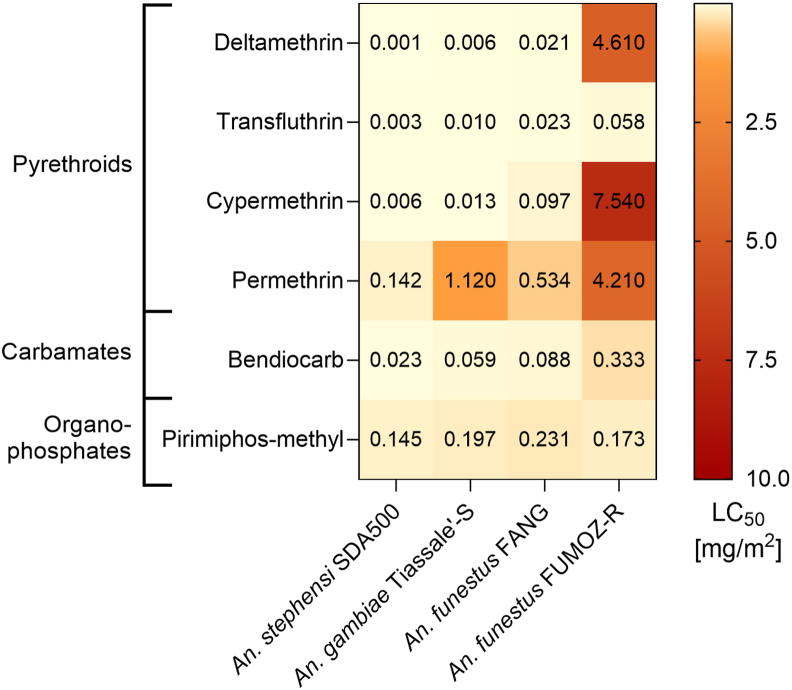
Heatmap showing LC_50_ values of all *Anopheles* strains included in the study, obtained through glazed tile bioassays.

The glazed tile bioassay and LC_50_ evaluations were repeated on adults of *An. funestus* (FANG and FUMOZ-R strains) and *An. gambiae* (Tiassalé-S strain) (Fig. 4; Supplementary Table S3). Note that results for *An. funestus* include data from Nolden et al. (2021) – performed with the same strains and methods – for α-cypermethrin, deltamethrin, cis-permethrin, permethrin, trans-permethrin, and transfluthrin. For the susceptible strains FANG and Tiassalé-S, deltamethrin proved the most toxic compound (FANG LC_50_ = 0.0206 mg/m^2^; Tiassalé-S LC_50_ = 0.00596 mg/m^2^). Excluding permethrin enantiomers, permethrin was the least effective compound (FANG LC_50_ = 0.543 mg/m^2^; Tiassalé-S LC_50_ = 1.12 mg/m^2^); the racemic mixture (61.5% trans-permetrhin, 30.3% cis-permethrin) had LC_50_ values higher than the cis-enantiomer and lower than the trans-enantiomer for both strains. As expected, the pyrethroid-resistant *An. funestus* FUMOZ-R strain showed very different LC_50_ values compared to the susceptible strains, with only transfluthrin and pirimiphos-methyl having comparable toxicity; all other compounds had LC_50_ values significantly higher than SDA500, Tiassalé-S and FANG samples.

The DDs proposed for bottle assays for *Anopheles* species were adapted to glazed tiles bioassays by conversion to mg/m^2^, maintaining the same mg a.i./surface area ratios and LC_50_ values were used to predict lethality at the DD values (Table 4). Besides permethrin, resulting always in resistant classification, the other compounds were ranked as susceptibility (lethality > 98%) and suspected resistance (lethality > 90%) for Tiassalé-S and FANG strains; FUMOZ-R manifested resistance for all the tested compounds, besides pirimiphos-methyl (suspected resistance).

**Table 4:** Evaluation of susceptibility through glazed tile assay by adapting discriminating doses from bottle assay.

Compound	*An. gambiae* Tiassalé-S			*An. funestus* FANG			*An. funestus* FUMOZ-R		
	DD (mg/m^2^)	Predicted lethality (%)	S/R	DD (mg/m^2^)	Predicted lethality (%)	S/R	DD (mg/m^2^)	Predicted lethality (%)	S/R
α-cypermethrin	0.446	98.1	S	0.446	98.5	S	0.446	0.52	R
Deltamethrin	0.446	100	S	0.446	98.6	S	0.446	7.59	R
Permethrin	0.767	41.4	R	0.767	68.7	R	0.767	3.27	R
Transfluthrin	0.143	99.6	S	0.143	93.6	SR	0.143	81.1	R
Bendiocarb	0.446	99.8	S	0.446	96.3	SR	0.446	66.5	R
Pirimiphos-methyl	0.714	91.7	SR	0.714	90.0	SR	0.714	90.6	SR

*Abbreviations*: DD, discriminating dose; S, susceptible; R, resistant; SR, suspected resistance.

## 4 Discussion

In the absence of previous studies on the resistant status of the SDA500 strain of *An. stephensi*, the colony reared in Envu (Monheim, Germany) since 2022, was rewarded as insecticide-susceptible after performing glazed tiles bioassays, bottle bioassays and larval bioassays. WHO bottle bioassay was the main method to confirm the susceptibility status, given the availability of reference DDs (bendiocarb, clothianidin, deltamethrin) (Vatandoost et al., 2019; WHO, 2022b; Corbel et al., 2023). The DD for clothianidin (0.01 mg/ml), to be tested with 800 ppm MERO®, only produced 71.8% lethality; increased MERO® concentration (2000 ppm) corresponded to increased levels of mortality, up to 97.7%. The transposition of the bottle assays’ DDs glazed tiles assays produced mortality levels regarded as susceptible for bendiocarb (99.3%), deltamethrin (99.7%) and transfluthrin (94.4%), while clothianidin data reached 11.6% only with the highest MERO® concentration (2000 ppm). Besides clothianidin, that showed very low levels in the glazed tiles, the other three compounds performed in a similar way adapting the DDs provided for the bottle assay, highlighting the possibility to simply use those values in the absence of reference doses for the glazed tile assay.

Larval bioassays confirmed the susceptibility of *An. stephensi* SDA500 in immature stages, although lethal concentrations available in literature for the species were very heterogeneous. Toxicity of deltamethrin on a susceptible *An. stephensi* laboratory strain, originally from Malaria Research Center, Chennai, India, resulted in LC_50_ = 0.0045 ppm, 3.1 times lower than the toxicity shown on SDA500 strain (LC_50_ = 0.014 ppm) (Gayathri and Balakrishna Murthy, 2006). Larval bioassays with permethrin, performed on susceptible and pyrethroid-resistant *An. stephensi* mosquitoes, resulted in LC_50_ = 0.0167 ppm on the susceptible Beech strain, about 10 times higher than the LC_50_ obtained on Envu’s SDA500 strain (LC_50_ = 0.102 ppm); on the other hand, the pyrethroid-resistant strain tested in the study (DUB-R) had an LC_50_ = 3.04 ppm, 30 times higher than SDA500 (Enayati et al., 2003). A more recent study from Negri et al. (2019) reported higher LC_50_ for permethrin on susceptible *An. stephensi* larvae from a laboratory strain reared in Italy (LC_50_ = 0.072 ppm), only 1.42-fold lower than the LC_50_ presented in this study. The organophosphate temephos showed an LC_50_ = 0.009 ppm on F2 larvae obtained from field mosquitoes from Sri Lanka (Jude et al., 2023), following the same range of toxicity obtained in this study (LC_50_ = 0.012 ppm).

Insect growth regulators (IGR) performed effectively against SDA500 larvae. Although the lack of toxicity data in the literature for this species, EI_50_-values can be found for other mosquitoes, such as *An. gambiae* larvae from the Kisumu susceptible strain (EI_50_ = 0.000088 ppm) (Jude et al., 2023) and *Ae. aegypti* larvae from the Rockefeller susceptible strain (EI_50_ = 0.00052 ppm) (Andrighetti et al., 2008). The low EI_50_ values reported in these studies, together with the results found in the present research, point out the efficacy in reducing mosquito population sizes through emergence inhibition, adding more value to IGRs as key candidates for *An. stephensi* larval control in African countries, as already suggested by Killeen et al. (2002).

Regarding glazed tile bioassays, no DDs are available in the literature, and no LC_50_ values were ever reported for *An. stephensi*, hence the LC_50_ values obtained through glazed tiles in this study were compared to bottle bioassay ones, showing promising insights for the adoption of the second technique in insecticidal screenings, given its advantages regarding drying time, material costs and adaptability on different surfaces. Despite its advantages, the number of individuals per replicate (*n* = 10) compared to the number of the bottle assay (*n* = 25) represents an important limitation for any possible validation.

The correlation between glazed tile and bottle assays showed significant results only when excluding the outliers (clothianidin, flupyradifurone, and imidacloprid), the application of whose requires the surfactant MERO® to prevent a.i. crystallization and to guarantee a proper coating of the treated surfaces (WHO, 2022b; Corbel et al., 2023). Each test showed issues with reproducibility and consistent results, which were already reported by the WHO in a multi-laboratory study to determinate DDs for bottle assays: imidacloprid did not manage to reach a plateau of mortality through concentration increase; on the other hand, clothianidin and flupyradifurone were reported as inconsistent because of the difficulty in homogeneously coating the bottles and of the volatility of the compounds (WHO, 2022a).

In our study, the ranks of toxicity resulting from the two techniques were very similar, besides neonicotinoids and flupyradifurone showing very different toxicity between the techniques. The three active ingredients dissolved in acetone + MERO® showed higher efficacy in the bottles than in the glazed tiles, highlighting a possible bias due to the surfactant. The WHO generally suggests concentrations of MERO® in a range of 200–1500 ppm, with 200 ppm for *An. albimanus*, 800 ppm for *Anopheles* spp., and 1500 ppm for *Aedes* spp., based on previous experiments that determined sublethal concentrations of the surfactant (Corbel et al., 2023). In our study, higher concentrations of MERO® corresponded to higher levels of lethality in both the techniques; considering the gap between the two methods, the lowest concentration of MERO® tested with clothianidin in the bottles (200 ppm) reached almost the highest concentration of MERO® in the glazed tiles (0.646 mg/m^2^ *vs* 1.06 mg/m^2^, respectively). An adaptation of the levels of MERO® could be considered to permit comparability of glazed tiles and bottle assays, but this will require further investigation.

The cause of the bias in active ingredients requiring MERO® was investigated through assays aimed to identify factors differing between bottles and glazed tiles. The material of the treated surface did not influence the results, also because both glass and glazed ceramic are non-porous and chemically inert materials. Another potential cause was the heterogeneity of the coating between the techniques, since the continuous rolling of the bottles may guarantee a more homogenous coating than the fast and manual coating of the tiles. Glazed tiles have been coated in the past through spraying of insecticide solutions *via* different techniques: airbrush connected to an automated track-sprayer (Wege et al., 1999), air compressor (Toews et al., 2003), and hand pump spray bottle (Jones and Bryant, 2012).

All these applications could guarantee a finely uniform coating of the tile; hence, the addition of the dispersant Atlox™ 3467 was thought to achieve a better coating, by improving the delivery of insecticide to mosquitoes on the tiles *via* making the a.i. remain homogenously suspended throughout the formulation. Atlox™ 3467 is among the dispersants recommended by the European Patent EP 1 926 380 B1 for the application of the neonicotinoid imidacloprid, usually in quantities around 100 g per suspension concentrate (SC) formulation (Neale and Patchett, 2014). Atlox™ produced different effects between clothianidin and flupyradifurone, reducing the efficacy of the first and enhancing the efficacy of the second both around three-fold rate. In general, the dispersant did not show a clear enhancement of compounds to which MERO® was added, indicating that, even though it may help to reach a more uniform coating and a finer fragmentation of the crystals of a.i., it is not directly involved in the lack of efficacy observed in the glazed tiles when using MERO®.

Given the low lethality rates for clothianidin coming from both bottle and tile bioassays, to exclude the possibility of a clothianidin-resistant phenotype, topical application was performed, and the resulting LC_50_ for clothianidin (0.0872 ng a.i./mg insect) was compared with data generated on *An. stephensi* for other neonicotinoids, given the absence of literature data for clothianidin itself: a strain susceptible to organochlorines, organophosphates and pyrethroids had LC_50_ values of 2.217, 0.295, and 0.946 ng a.i./mg insect for imidacloprid, thiacloprid and thiamethoxam, respectively (Uragayala et al., 2015). The topical application performed on SDA500 showed a LC_50_ value significantly lower than all the conditions tested by Uragayala et al. (2015), highlighting the clothianidin-susceptibility of the colony object of this study.

Synergist bioassays with PBO and triflumizole, inhibitors of mixed-function oxidase (MFO) activity (Brogdon and McAllister, 1998), excluded the presence of resistance mechanisms based on cytochrome P450s and other MFOs. The absence of significant difference between LC_50_ values of deltamethrin-treated mosquitoes (LC_50_ = 0.00118 mg/m^2^) and the two synergistic combinations of PBO and deltamethrin (LC_50_ = 0.000685 mg/m^2^) and triflumizole and deltamethrin (LC_50_ = 0.0017 mg/m^2^) excluded overexpression of P450 enzymes as enhancers of pyrethroids detoxification (metabolic resistance), as it is expected in susceptible colonies (Brogdon and McAllister, 1998; Brooke et al., 2001).

Although no reference data are available from the literature for glazed tiles assays on *An. stephensi*, the technique was previously used to characterise the toxicity levels of several active ingredients on *An. funestus* FANG (susceptible) and FUMOZ-R (pyrethroid-resistant) strains by Nolden et al. (2021). The panel of active ingredients tested in that study has been implemented with bendiocarb and pirimiphos-methyl, expanding the research to different classes than pyrethroids (carbamates and organophosphates, respectively). All compounds previously tested by Nolden et al. (2021) have been tested on *An. stephensi* SDA500 and *An. gambiae* Tiassalé-S strains as well. Adapting DDs from the bottle assay to glazed tiles, it was possible to observe similar results among the susceptible strains, excluding permethrin, for which the DD always resulted in resistant classification. Since this was the case for all strains, this seems most likely to be related to an aspect of the methodology. An issue with drying time seems an unlikely explanation since the same procedure produced expected results for other insecticides. The DD for the WHO bottle bioassay is based on a racemic mixture of trans-permethrin and cis-permethrin with a ratio included between 75:25% and 60:40%; in this study, we used a batch with a ratio falling within the WHO limits (> 60% trans-). However, we cannot exclude the possibility that the results may have been influenced by a slight variation in the ratio of the two enantiomers, which have different toxicities (Supplementary Table S3). On the other hand, the agreement among the results of this study seems to suggest the possibility of adopting the same DDs for, at least, *An. funestus*, *An. gambiae* and *An*. *stephensi*, and potentially for the whole genus *Anopheles*.

## 5 Conclusions

A comprehensive characterization of the key reference strain SDA500 of *An. stephensi*, for its susceptibility to multiple insecticides, including both traditional and alternative active ingredients, provides an important baseline resource for future studies, which will be crucial to identify alternative control approaches for *An. stephensi*. The glazed tile bioassay, a high-throughput technique for contact insecticides presenting operational advantages compared to standard WHO methods, has shown promising results, suggesting a role in screening procedures for mosquito insecticides. However, for it to become a fully recognised alternative to bottle bioassays, a multi-centre study, following WHO validation frameworks, would be required to formally establish technique-specific diagnostic doses. Not only useful to validate the glazed tile bioassay, but also important for insecticidal screening purposes, the comparison with other anopheline strains added important information about the variability of toxicity levels of active ingredients toward species of the same genus. For some insecticides for which reference data may be unavailable for other *Anopheles* species, the LC_50_ values presented here might be useful as an interim comparator to validate susceptibility or detect emergent resistance, helping to identify effective insecticidal compounds for control of multiple malaria vectors.

## Ethical approval

Not applicable.

## CRediT authorship contribution statement

**Michele Matera:** Conceptualisation, Formal analysis, Investigation, Methodology, Visualisation, Writing – original draft, Writing – review & editing. **Melanie Nolden:** Methodology, Resources, Supervision. **Sebastian Horstmann:** Conceptualisation, Resources, Supervision, Writing – review & editing. **Derric Nimmo:** Conceptualisation, Funding acquisition, Supervision. **Mark J.I. Paine:** Supervision, Writing – review & editing. **David Weetman:** Conceptualisation, Data curation, Funding acquisition, Project administration, Resources, Supervision, Validation, Writing – review & editing.

## Funding

The project was funded by Envu, 2022 ES Deutschland GmbH and the Innovative Vector Control Consortium (IVCC).

## Declaration of competing interests

The authors declare that they have no known competing financial interests or personal relationships that could have appeared to influence the work reported in this paper. Given their role as Co-Editor, David Weetman had no involvement in the peer review of this article and has no access to information regarding its peer review. Full responsibility for the editorial process for this article was delegated to Professor Aneta Kostadinova (Editor-in-Chief).

## Data Availability

All data generated or analysed during this study are included in this published article and its supplementary file. Raw data are available from the corresponding author upon request.
