# Supplementary material for: Susceptibility of Anopheles stephensi SDA500 strain to common insecticides and efficacy of glazed tile bioassay for resistance characterization
Source: Curr Res Parasitol Vector Borne Dis. 2026 Jan 1;9:100347. doi: 10.1016/j.crpvbd.2026.100347 (PMC12811468; doi:10.1016/j.crpvbd.2026.100347)
Supplement: Multimedia component 1 [file mmc1.pdf]

Supplementary materials

Supplementary Table S1. Dose-mortality data (24 h) for insecticidal active ingredients and synergists against adult female *An. stephensi* (SDA500 strain) in glazed tile bioassays.

Compound	LC_{50} [mg/m^2]	95% CI	Slope	95% CI	n
deltamethrin	0.00118	0.000822-0.00165	1.35	0.9-3.88	720
transfluthrin	0.00340	0.00198-0.00554	0.927	0.624-1.43	480
permethrin	0.142	0.122-0.164	1.80	1.38-3.10	480
cis-permethrin	0.0462	0.0395-0.0548	1.91	1.45-3.08	240
trans-permethrin	0.845	0.618-1.14	1.43	0.965-3.7	240
α-cypermethrin	0.00555	0.0047-0.00648	1.50	1.21-1.98	240
4'OH deltamethrin	0.173	0.14-0.214	1.61	1.18-3.03	240
4'OH permethrin	9.37	6.24-14.02	1.69	0.998-3.14	240
bendiocarb	0.0234	0.0185-0.0289	1.69	1.22-4.05	480
pyrimiphos-methyl	0.145	0.128-0.162	1.93	1.52-3.29	240
clothianidin	1.06	0.951-1.186	1.87	1.54-2.45	240
imidacloprid	4.96	4.46-5.58	2.08	1.61-4.26	480
flupyradifurone	348.0	123-2302	0.413	0.245-0.661	480
PBO	575.4	351.3-1504	0.878	0.496-1.56	240
triflumizole	52418	-	0.487	0.0977-	240

Abbreviations: LC_{50}: Lethal Concentration 50; 95% CI: 95% Confidence Interval; n: number of mosquitoes in the test.

Supplementary Table S2. Log-dose mortality data for insecticidal active ingredients against larval *An. stephensi* (SDA500 strain).

Compound	LC_{50} / EI_{50}* [ppm]	95% CI	Slope	95% CI	n
triflumuron*	0.00108*	0.000839-0.00138	1.59	1.17-2.46	960
diflubenzuon*	0.00208*	0.0018-0.00243	1.9	1.44-3.26	480
pyriproxyfen*	0.003*	0.0022-0.00406	1.17	0.868-1.63	960
abamectin	0.0203	0.0146-0.0287	1.11	0.852-1.56	540
bendiocarb	1.1	1.032-1.19	2.16	1.72-4.88	960
deltamethrin	0.0138	0.012-0.0154	2.72	2.31-3.59	480
permethrin	0.102	0.0955-0.112	3.58	3.27-4.08	480
pirimiphos-methyl	0.0125	0.00909-0.0167	1.54	1.07-2.49	480
temephos	0.0123	0.0104-0.0147	1.9	1.37-3.09	480

Abbreviations: LC_{50}: Lethal Concentration 50; 95% CI: 95% Confidence Interval; n: number of mosquitoes in the test. *Efficacy of IGRs (triflumuron and pyriproxyfen) evaluated as Emergence Inhibition (EI) after the full development into adults of all alive individuals.

Supplementary Table S3. Dose-mortality data (24 h) after contact exposure (30 min) in glazed tile assay for different *Anopheles* strains: *An. stephensi* SDA500 susceptible strain, *An. gambiae* Tiassalé-S susceptible strain, *An. funestus* FANG susceptible strain*, An. funestus* FUMOZ-R pyrethroid-resistant strain.

Strain	SDA500		Tiassalé-S		FANG		FUMOZ-R	
**Compund**	LC_{50} [mg/m^2]	95% CI	LC_{50} [mg/m^2]	95% CI	LC_{50} [mg/m^2]	95% CI	LC_{50} [mg/m^2]	95% CI
deltamethrin	0.00118	0.000822-0.00165	0.00596	0.00466-0.00745	0.0206	0.0153-0.0273	4.61	2.73-7.5
transfluthrin	0.0034	0.00198-0.00554	0.0104	0.00782-0.0128	0.023	0.0155-0.0324	0.0576	0.0191-0.112
α-cypermethrin	0.00555	0.0047-0.00648	0.0134	0.0101-0.018	0.0968	0.0686-0.0126	7.54	5.24-10.4
permethrin	0.142	0.122-0.164	1.12	0.732-1.76	0.543	0.409-0.702	4.21	2.79-5.88
cis-permethrin	0.0462	0.0395-0.0548	0.883	0.764-1.02	0.154	0.096-0.228	1.6	0.933-2.63
trans-permethrin	0.845	0.618-1.14	1.98	1.39-2.8	1.41	1.04-1.92	9.24	6.24-13.8
bendiocarb	0.0234	0.0185-0.0289	0.0593	0.0515-0.0657	0.0877	0.0691-0.112	0.333	0.268-0.411
pirimiphos-methyl	0.145	0.128-1.19	0.197	0.159-0.23	0.231	0.197-0.275	0.173	0.139-0.213

Abbreviations: LC_{50}: Lethal Concentration 50; 95% CI: 95% Confidence Interval.

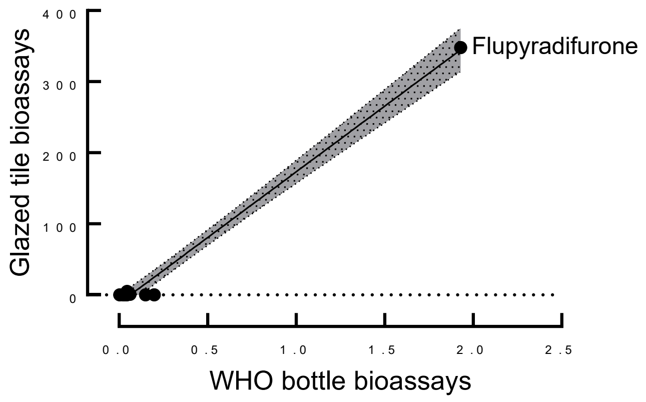

Supplementary Figure S1. Comparison of WHO bottle bioassays and glazed tile bioassays. Plot of mean LC_{50} values from WHO bottle bioassays and glazed tile bioassays including all insecticides, highlighting flupyradifurone (outlier).

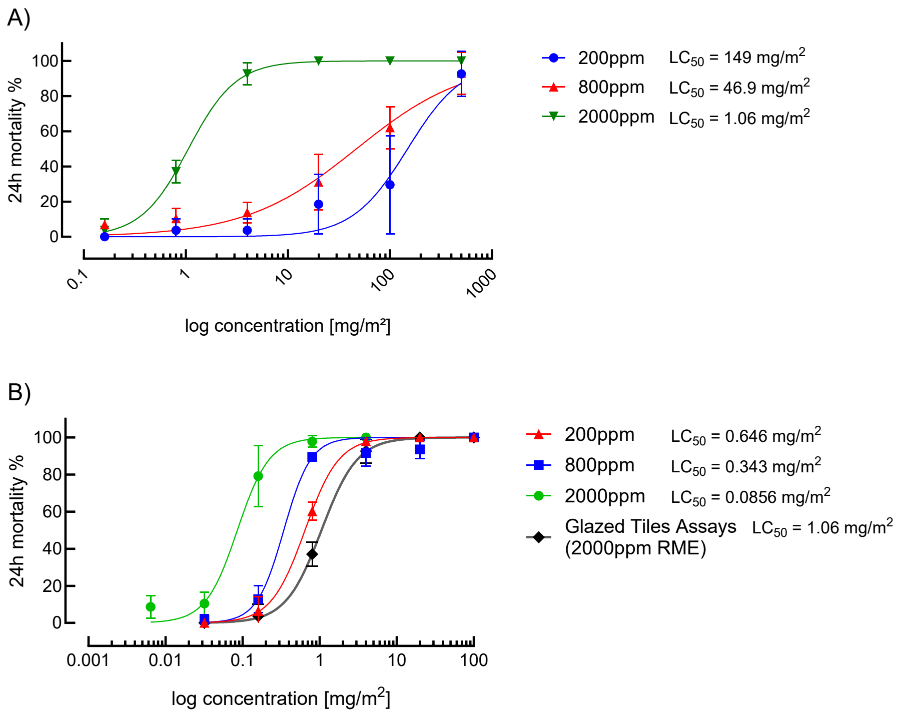

Supplementary Figure S2. A) LC_{50} curves for clothianidin from glazed tile bioassays with three concentrations of MERO®: 200ppm, 800ppm, 2000ppm. B) LC_{50} curves for clothianidin obtained through bottle assays comparing three concentrations of MERO (200ppm, 800ppm, 2000ppm) and results from glazed tiles with the most effective MERO® concentration (2000ppm).

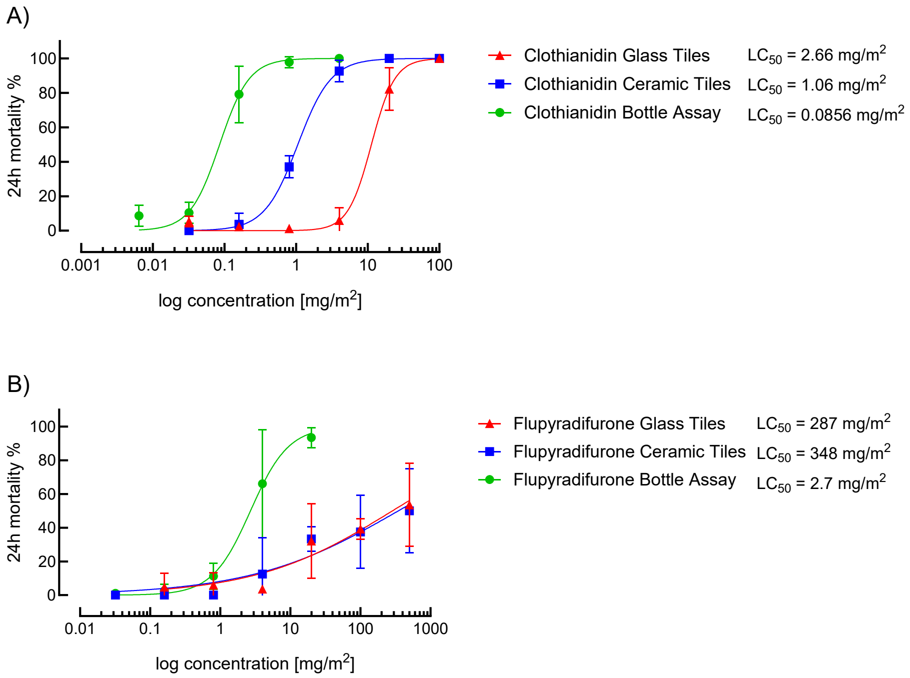

Supplementary Figure S3. LC_{50} curves for clothianidin (A) and flupyradifurone (B) comparing glass tiles, ceramic tiles and bottle assays.

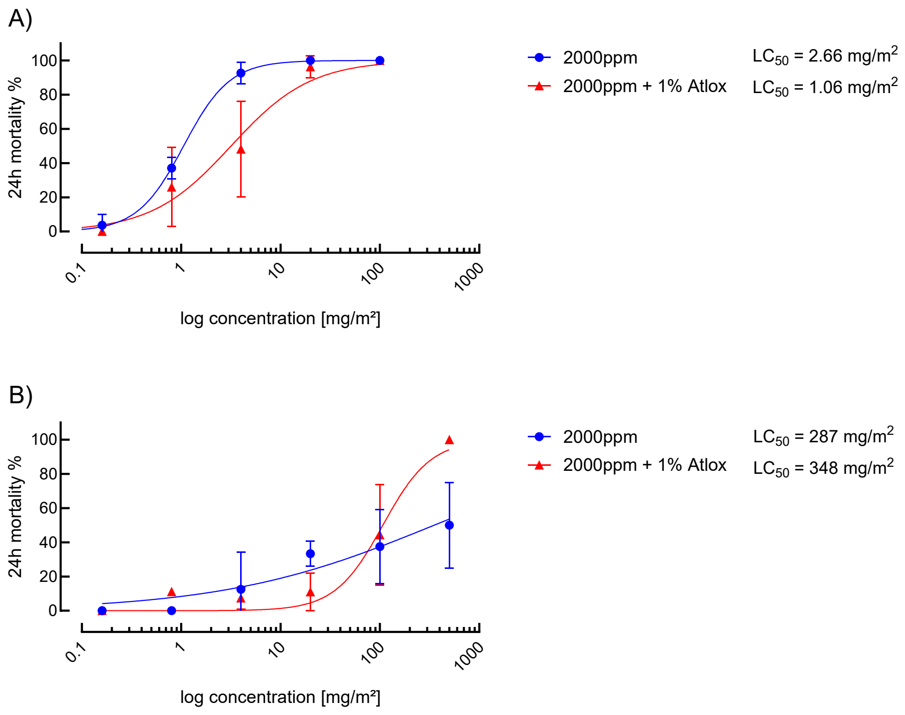

Supplementary Figure S4. LC_{50} curves for clothianidin (A) and flupyradifurone (B) from glazed tile assays comparing 2000ppm MERO® with and without 1% Atlox™ 3467.

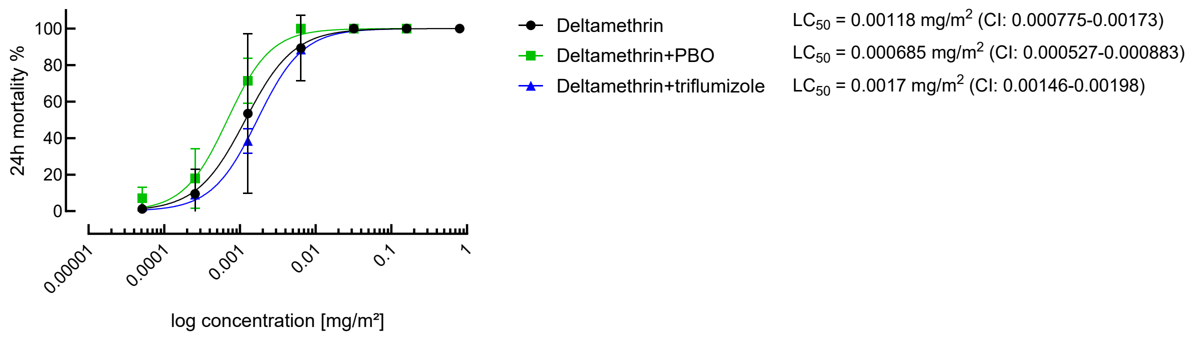

Supplementary Figure S5. Dose-response curves of synergistic assays: glazed tiles bioassays with pre-application of PBO and triflumizole followed by deltamethrin, compared to deltamethrin alone. CI: 95% confidence interval.
